# Fatigue Fracture Analysis on 2524 Aluminum Alloy with the Influence of Creep-Aging Forming Processes

**DOI:** 10.3390/ma15093244

**Published:** 2022-04-30

**Authors:** Liyong Ma, Chi Liu, Minglei Ma, Zhanying Wang, Donghao Wu, Lijuan Liu, Mingxing Song

**Affiliations:** 1 School of Mechanical Engineering, Hebei University of Architecture, Zhangjiakou 075031, China; maliyong@buaa.edu.cn (L.M.); wzx2124@hebiace.edu.cn (D.W.); zyq1292@hebiace.edu.cn (L.L.); 2 School of Mechanical and Electrical Engineering, Changsha University, Changsha 410199, China; liuchi@ccsu.edu.cn; 3 Zhangjiakou Cigarette Factory Co., Ltd., Zhangjiakou 075001, China; why2048@hebiace.edu.cn; 4 Zhangjiakou Special Equipment Intelligent Monitoring Operation and Maintenance Technology Innovation Center, Zhangjiakou 075031, China

**Keywords:** creep-aging forming, creep-aging temperature, creep stress, 2524 aluminum alloy, fatigue-crack propagation rate, fatigue fracture morphology

## Abstract

The different creep-aging forming processes of 2524 aluminum alloy were taken as the research object, and the effects of creep-aging temperature and creep stress on the fatigue-crack propagation properties of the alloy were studied. The research results showed the following under the same sintering time of 9 h, at creep-aging temperatures of 100 °C, 130 °C, 160 °C, and 180 °C, respectively, with an increase in creep-aging temperature: the fatigue-crack propagation rate was promoted, the spacing of fatigue striations increased, and the sizes of dimples decreased while the number was enlarged; this proves that the fatigue property of the alloy was weakened. Compared with the specimens with creep deformation radii of 1000 mm and 1500 mm, the creep deformation stress was the smallest when the forming radius was 1800 mm, with a higher threshold value of fatigue-crack growth in the near-threshold region of fatigue-crack propagation (Δ*K* ≤ 8 MPa·m^1/2^). Under the same fatigue cycle, the specimens under the action of larger creep stress endured a longer fatigue stable-propagation time and a faster fracture speed. Comparing the effect of creep-aging temperature and creep stress, the creep-aging temperature plays a dominant role in the fatigue-crack propagation of creep-aged 2524 aluminum alloy.

## 1. Introduction

2524 aluminum alloy is a new type of high-strength aluminum alloy for aviation [1] that is mainly used for wing skins [2]. During service, it is subjected to loads in complex environments, so its fatigue performance is particularly critical [3]. Compared with aluminum alloys such as 2024, 2124 and 2224, 2524 aluminum alloy has higher strength and better fatigue properties [4,5]. The fracture toughness is increased by 15~20%, the fatigue resistance doubles, and the fatigue life is increased by 27~45% [6,7], which is well suited to the requirements of modern aircraft design for material damage tolerance [8]. Meanwhile, with the maturity of the creep-aging forming process, combined with the good hot-working properties of 2524 aluminum alloy, the application prospects of 2524 aluminum alloy in the future aerospace industry are broad [9].

During the creep-aging forming process, the microstructure and evolution process of the material are complex, and the factors that affect the fatigue properties of the creep-aging forming material also become complicated. Pitcher et al. [10] studied creep-aging-formed 2024A, 8090, and 7449 aluminum alloys from the two aspects of springback and damage tolerance, and conducted a large number of fatigue-crack propagation rate experiments. The result showed that 2024A and 8090 had sufficient damage resistance after creep deformation, which can be useful for making lower wing skins. Rafiq A. Siddiqui et al. [11] studied the effects of age temperature and duration on the fatigue properties of 6063 aluminum alloys. The study found the vacancy diffusion mechanism played a significant role in the formation of Guinier–Preston (GP) zones, in which the solid solution is precipitated due to uniform nucleation during the dissolution process, and a fine quasi-stable phase is precipitated. The structure of this stable phase was similar to that of the main matrix, and the two were coherent with each other, thus disturbing the regularity of the lattice, leading to the increase in the fatigue defects of the alloy. Brav G.H. et al. [12] studied the effects of different aging processes on the crack propagation rate of the 2024-T351 alloy and Al–Cu–Mg–Li alloy. The results showed that artificial aging increases the strength of the alloy, but it also reduces the crack propagation resistance of the alloy. In addition, with the prolongation of artificial aging duration, the crack propagation rate increased monotonically. The authors believed that the reduction in crack propagation resistance was related to the precipitation of the T1 phase and *S′* phase in the alloy; however, the influence mechanism of the aging process on the precipitation phase was not discussed, nor was the effect of fine precipitation on the crack-tip slip-mode. Sarioglu F. and Orhaner F.Ö. [13] researched the fatigue property of the 2024-T3 aluminum alloy after aging at a low temperature of 130 °C for 100 h/1000 h. The results showed that long-term aging eliminated the difference in the crack propagation rate of alloys in the sampling directions (L-T, T-L, 60°). Burba et al. [14] found that the minimum fatigue life of the alloy was mainly affected by the density of the θ′ precipitation phase, but had little effect on the average fatigue life. Yang [15] conducted a lot of research on the effect of the creep-aging forming process on the high-cycle fatigue properties of Al-Zn-Mg series-7075 high-strength aluminum alloys. The study found that the increase in creep-aging temperature and time has a positive effect on the improvement of alloy fatigue properties. The effect of preloading stress will reduce the fatigue resistance of the alloy. However, due to the differences in microstructure and chemical composition, the conclusions of this study were not applicable to 2524 aluminum alloys.

Xu et al. [16] studied the effect of tensile pre-strain before creep-aging forming on the mechanical properties of 2524 aluminum alloy using a constant-stress creep-aging test. they found that the magnitude of creep strain was greatly increased with the increase in pre-strain and that the pre-strain improved the formability, mechanical properties and microstructures of 2524. In addition, Xu et al. [17] also conducted research on the tension and compression creep-aging behaviors of Al-Cu-Mg alloy. They demonstrated that the creep strains under tensile stresses were larger than the creep strains under compressive stresses. Meanwhile, the formation of the *S* phase in the aluminum matrix was caused by the compressive stress, inhibiting the precipitates in the grain boundary, which was beneficial to the hardness of the compression creep-aged alloy. Liu et al. [18] studied the effects of creep-aging and artificial aging on the fatigue-crack propagation of 2524 aluminum alloy. They found that the prolonged aging time resulted in excessive precipitation of the needle-like *S*′ phase, changed the dislocation slip mode, reduced the reversibility of the slip, and accelerated the accumulation of fatigue damage. In addition, creep stress accelerated the aging precipitation process of the alloy. Compared with artificial aging, under the same aging time, the size of the precipitates in the creep-aged alloy was larger and the yield strength and hardness were increased, but the fatigue resistance was decreased. In our previous work [19], we studied the effects of creep time on the microstructural and mechanical properties, as well as the fatigue-crack propagation, in 2524 aluminum alloy. TEM was employed for the observation of precipitation and dislocation.

Most scholars have focused on the relationship between the creep/stress relaxation mechanism and the material deformation. However, the relationship between creep-aging temperature, creep stress, and the fatigue properties of materials has not been comprehensively and systematically studied.

This paper will focus on analyzing the macro-microscopic characteristics of the fatigue-crack fractures of creep-formed specimens, and analyze the fracture mode, crack propagation path and fracture mechanism of creep-formed components. The fatigue property of creep-formed alloys at different creep-aging temperatures and creep stresses will be explored from the micro-morphological features.

## 2. Experimental Processes

### 2.1. Materials

In this study, a 2524 aluminum alloy sheet for aviation (the thickness was 3.5 mm) was selected for research. After the alloy was solution-treated, it was cold-machined, and then naturally aged to a stable state. The main chemical composition of 2524 aluminum alloy is shown in Table 1 [9,16].

### 2.2. Creep-Aging Forming

**Figure 1:**
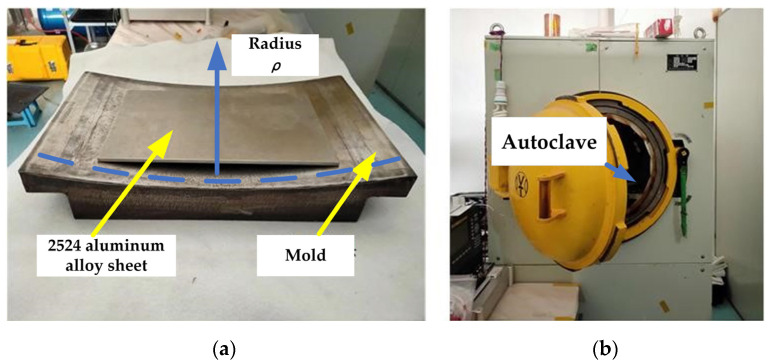
Creep-aging forming mold and tooling: (**a**) mold and sheet; (**b**) autoclave.

The creep-aging forming experiment specimen and mold are shown in Figure 1a, and the size of the experiment sheet was 360 mm × 220 mm. The 2524 aluminum alloy sheet was placed in the center of the mold, then the mold and sheet were wrapped with air felt and a vacuum bag; then, the bag was sealed with heat-resistant glue. The bag was gradually evacuated to a vacuum, and a negative pressure of 0.1 MPa was maintained. The sheet was elastically deformed under uniform load until it was completely fitted with the mold surface. The deformed sheet and mold were put into an autoclave (Figure 1b), with a heating rate of 1.5 °C/min. In addition, the creep-aging treatment was carried out according to the set creep-aging temperature, and the vacuum bag was kept in a sealed state during the creep-forming process. After the set aging time was reached, the load and temperature were removed, and the components were cooled in a furnace, obtaining the final desired shape.

(1) To study the effect of creep-aging temperature on fatigue property, creep deformation experiments were carried out at creep-aging temperatures of 100 °C, 130 °C, 160 °C and 180 °C, respectively, and the creep-aging time of all specimens was 9 h.

(2) To study the effect of creep stress, the radii of curvature *ρ* were 1000 mm, 1500 mm and 1800 mm, respectively. The larger the radius *ρ*, the smaller the creep stress. The creep-aging temperature was 160 °C, and the creep time was 9 h.

### 2.3. Fatigue Experiment

**Figure 2:**
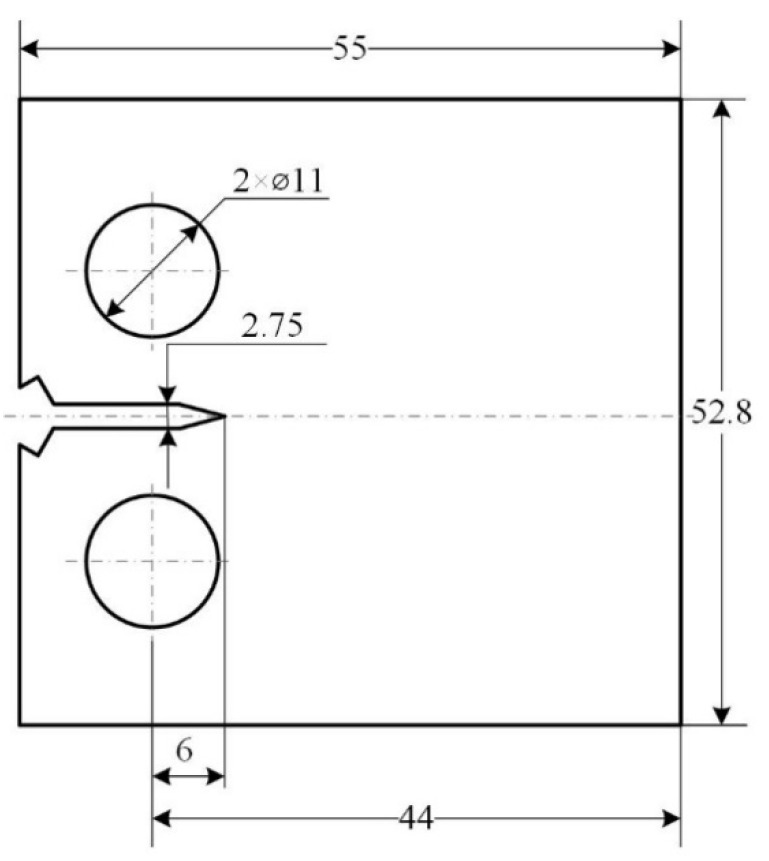
Schematic diagram of CT specimen for fatigue-crack propagation experiment.

The fatigue-crack propagation experiment was carried out at room temperature. The sample preparation and stress loading method of the fatigue-crack propagation specimen were designed according to ASTM-E647 [20]. The experimental equipment was the MTS-810 fatigue experimenting machine made in the U.S. The fatigue-crack propagation experiment utilized standard compact tensile specimens (CT specimens), and the specimens were cut using a wire electric discharge. The size of the sample is shown in Figure 2. Before the fatigue-crack propagation experiment, a crack of 2.75 mm was prefabricated using the pre-crack module in the experiment system. The experiment adopted sine wave loading, with the maximum loading of 2400 N. The stress ratio *R* of 0.1~0.5 (*R* = *P*_min_/*P*_max_), the loading frequency *f* of 10 Hz, and the crack length *a* were detected by the compliance control method (COD).

The fatigue-crack propagation data obtained in the experiment were processed using the seven-point incremental polynomial method. For data point *i* and its front 3 points and back 3 points—a total of 7 continuous data points—the quadratic polynomial was employed to perform local fitting and derivation, and the fitting values of the fatigue-crack propagation rate were obtained using Equation (1):

(1) $$a_{i}=b_{0}+b_{1}\left[\frac{N_{i}-C_{1}}{C_{2}}\right]+b_{2}{\left[\frac{N_{i}-C_{1}}{C_{2}}\right]}^{2}$$

where *N_i_* is the number of cycles, *a_i_* is the fitting crack length value; *b*_0_, *b*_1_ and *b*_2_ are the regression parameters determined according to the minimum squared deviation between the observed value of the crack length and the fitting value; *C*_1_ = (*N_i−3_* − *N_i+3_*)/2; *C*_2_ = (*N_i+3_* − *N_i−3_*)/2; and −1 ≤ (*N_i−3_* − *C*_1_)/*C*_2_ ≤ 1. By derivation of Equation (1), the crack propagation rate at *N_i_* can be obtained:

(2) $${\left(\frac{da}{dN}\right)}_{a_{i}}=\frac{b_{1}}{C_{2}}+\frac{2b_{2}(N_{i}-C_{1})}{C_{2}^{2}}$$

For CT specimens with type-I open cracks, the range of stress intensity factor Δ*K* at the crack tip can be calculated using Equation (3) [19,20]:

(3) $$\Delta K=\frac{\Delta P}{B\sqrt{W}}\cdot \frac{\left(2+\alpha \right)}{{\left(1-\alpha \right)}^{3/2}}(0.886+4.64\alpha -13.32\alpha^{2}+14.72\alpha^{3}-5.6\alpha^{4})$$

where Δ*P* is the force value range; Δ*P = P*_max_ − *P*_min_, *P*_max_ is the maximum loading force; *P*_min_ is the minimum loading force; *α* = *a*/*W*, *a* is the crack length; *B* is the width of the specimen (*B* = 5 mm); and *W* is the width of the specimen (*W* = 44 mm).

## 3. Result and Discussion

### 3.1. Effect of Creep-Aging Temperature on Fatigue-Crack Propagation

#### 3.1.1. Effect of Creep-Aging Temperature on Fatigue-Crack Growth Rate

**Figure 3:**
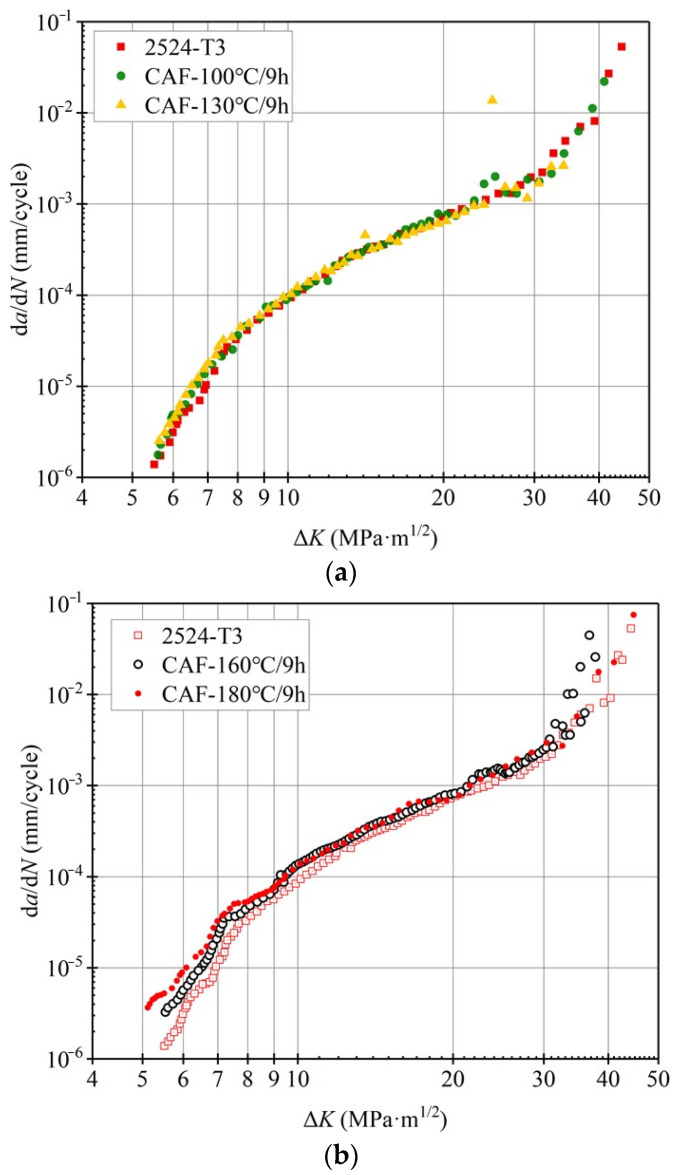
Fatigue-crack propagation rate d*a*/d*N*-Δ*K* curves of 2524 aluminum alloy at different creep-aging temperatures: (**a**) 100 °C and 130 °C; (**b**) 160 °C and 180 °C.

The creep-aging temperatures were 100 °C, 130 °C, 160 °C and 180 °C, respectively, and the aging time was the same, i.e., 9 h. The fatigue-crack propagation rate experiment was carried out. The d*a*/d*N* − Δ*K* curve is shown in Figure 3. In Figure 3a, the crack propagation curves of the creep-formed alloy at 100 °C and 130 °C coincide with those of 2524 aluminum alloy without creep forming, indicating a fatigue property with no significant change at lower creep-aging temperatures (<130 °C), because of insufficient effective deformation of the alloy at lower temperatures. Additionally, the d*a*/d*N* − Δ*K* curve of 2524-T3 aluminum alloy without creep forming was taken as the comparison data. 2524-T3 is a kind of alloy obtained through cold-working of 2524 aluminum alloy after solution treatment, then stabilization by natural aging.

Nevertheless, under the age temperatures of 160 °C and 180 °C, the crack propagation rate curves show significant differences (Figure 3b). In the near-threshold region (Δ*K* ≤ 8 MPa·m^1/2^), the crack propagation resistance of the alloy is significantly reduced under high-temperature creep-aging forming. Under the same Δ*K* of 6 MPa·m^1/2^, when the creep-aging temperature is 160 °C, d*a*/d*N* = 5.62 × 10^−6^ mm/cycles^−1^, but when the creep-aging temperature is 180 °C, d*a*/d*N* is increased to 9.11 × 10^−6^ mm/cycles^−1^, showing that the crack propagation resistance of 2524 aluminum alloy is decreased to a certain extent under high-temperature creep-aging forming. In addition, with the increase in Δ*K*, the difference in crack propagation rate gradually decreases. According to the curve, it is delineated in the Paris region of 10 MPa·m^1/2^ ≤ Δ*K* ≤ 30 MPa·m^1/2^, and the curve shows an obvious linear relationship.

The straight part of the d*a*/d*N* − Δ*K* curve in the double logarithmic coordinate is fitted by the Paris equation, and the corresponding fitting constants *C*, *n* and the fatigue-crack propagation rate under the same Δ*K* are shown in Table 2. The error value is 2.16%. The values of *n* are close, ranging from 2.7 to 3.0, indicating that the creep-aging temperature has little effect on the crack propagation rate of 2524 aluminum alloy in the medium and high stress range. When Δ*K* exceeds 30 MPa·m^1/2^, the curve of d*a*/d*N* − Δ*K* has an obvious turning point, and the crack propagation rate d*a*/d*N* increases rapidly from 10^−3^ mm/cycles to 0.1 mm/cycles until an instability fracture occurs.

#### 3.1.2. Fracture Morphologies at Different Creep-Aging Temperatures

(1) Fatigue-crack propagation zone

**Figure 4:**
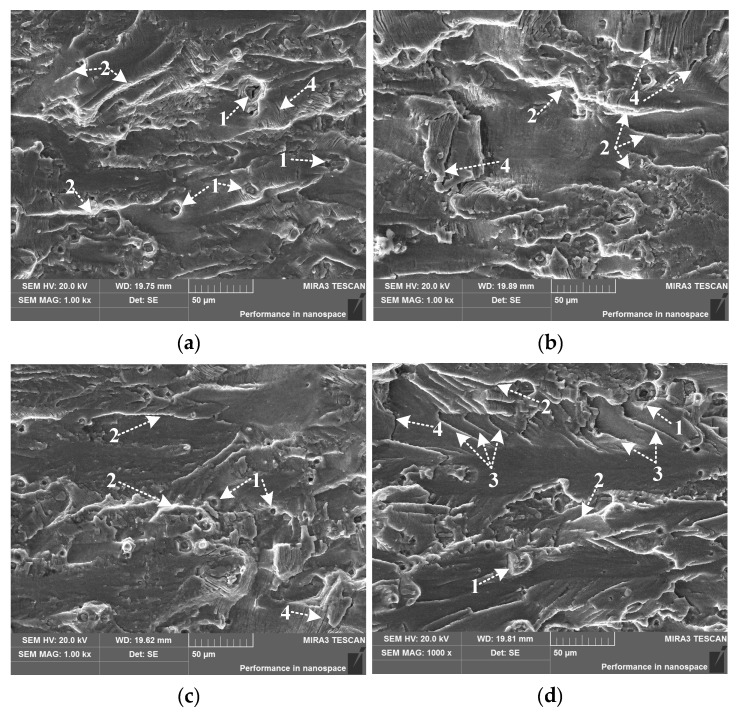
SEM images of stable fatigue-crack propagating zone at different creep-aging temperatures (*a* = 5 mm): (**a**) 100 °C—9 h; (**b**) 130 °C—9 h; (**c**) 160 °C—9 h; (**d**) 180 °C—9 h. Marks in the figure: 1—micropore; 2—tear edge; 3—shear edge; 4—secondary crack.

The creep-forming temperature has a decisive effect on the solute precipitation in the supersaturated state of 2524 aluminum alloy [21]. The increase in the age temperature enhances the atomic activity in the alloy, resulting in a quick increase in the precipitation rate of the precipitation phase [22]. Therefore, the nucleation, growth and enrichment of the precipitates during the aging process will be affected by the age temperature, which will lead to changes in the fatigue properties of the material. Figure 4 shows the fracture morphology of 2524 aluminum alloy at crack length *a* = 5 mm after aging at different creep-aging temperatures (100 °C, 130 °C, 160 °C, 180 °C) for 9 h. The corresponding Δ*K* at this time is about 15 MPa·m^1/2^, and the crack is in the stable propagating stage.

In Figure 4, the fatigue sections of the specimens are all flat, and the relative torsion of the cleavage planes in adjacent grains makes the crack propagate along many transgranular planes, forming smooth and flat sections, i.e., large fatigue platforms. These fatigue platforms are connected by the tearing edge, indicated by mark 2 in the figure. The tearing edge is deflected by an angle of 10°~40° relative to the main crack propagation direction, and the fracture shows the characteristics of a ductile transgranular fracture. There are also many micro-pores (mark 1) distributed on the cross-section, which originate from the tiny plastic deformations confined around the coarser second-phase particles during the fatigue process. Meanwhile, secondary cracks (mark 4) approximately perpendicular to the direction of the main crack-propagation plane are also observed, which propagate into the material. There are more fracture cleavage steps in the fracture morphology under high-temperature aging of 180 °C—9 h, and the fracture surface is rough, with part of the fracture morphology even showing slight brittle fracture characteristics. From Figure 4d, it can be seen that the obvious small fatigue steps are connected by the shear edges at mark 3; moreover, the heights are different, indicating that the aging precipitation and hardening rate of 2524 aluminum alloy are promoted due to high-temperature aging. The alloy enters the overaging state in advance, and the ductility decreases significantly, which adversely affects the fatigue property and toughness of the alloy.

**Figure 5:**
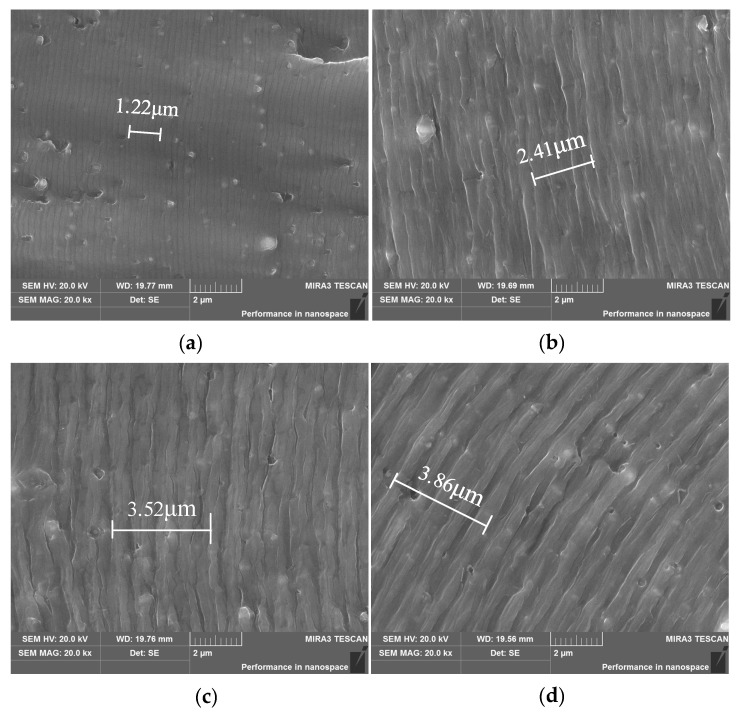
SEM images of fatigue striations in the stable propagation zone at different creep-aging temperatures (*a* = 5 mm): (**a**) 100 °C—9 h; (**b**) 130 °C—9 h; (**c**) 160 °C—9 h; (**d**) 180 °C—9 h.

The micro-morphology of the corresponding position in Figure 4 is magnified to 20,000 times for observation, and fatigue striations can be observed, as shown in Figure 5. There are obvious differences in the fatigue striation spacing of the specimens under different creep-aging temperatures. Since the spacing of only one fatigue striation is too small to measure, and the measured value of only one fatigue striation often brings in error, the spacing of five fatigue striations was measured for precision in mirroring the effect of creep temperature and stress on the fatigue-crack propagation. Under the low-temperature aging of 100 °C—9 h and 130 °C—9 h, the spacing of five fatigue striations are relatively small, at 1.22 μm and 2.41 μm, respectively, and the striation morphology is very clear and regular. With the increase in creep-aging temperature, under the same crack length, the striation spacing increases, and the appearance is rough. Under the high temperature aging of 160 °C—9 h and 180 °C—9 h, the average striation spacing is 3.52 μm and 3.86 μm. The difference in fatigue striation spacing reflects the increase in the size and volume fraction of the alloy precipitates in the peak or overaging state, which increases the strength of the material, but reduces its elongation and increases its brittleness, which makes fatigue streaks less likely to occur.

At different creep-aging temperatures, when the precipitated strengthening phase maintains a coherent or semi-coherent relationship with the matrix, it is generally believed that dislocations can cut through the precipitated phase [23], and then plane slip occurs, resulting in uneven deformation in local areas. As the slip plane continues to expand, the crack propagation path may deflect, kink, and bifurcate, reducing the rate of crack propagation. When the strengthening phase is incoherent with the matrix, the dislocations bypass the precipitation phase, and the deformation in the local area is relatively uniform; this reduces the possibility of deflection, kink and bifurcation during crack propagation, thereby increasing the rate of crack propagation. Therefore, for samples with an age temperature of 100 °C and 130 °C, the precipitates maintain a coherent or semi-coherent relationship with the matrix, and the fatigue-crack propagation rate is slow. However, when the age temperature is 180 °C, the precipitation phase of the material grows up and breaks away from the semi-coherent relationship with the matrix, so the crack propagation rate of the sample is at its fastest, as shown in Figure 3b.

(2) Fatigue fracture zone

**Figure 6:**
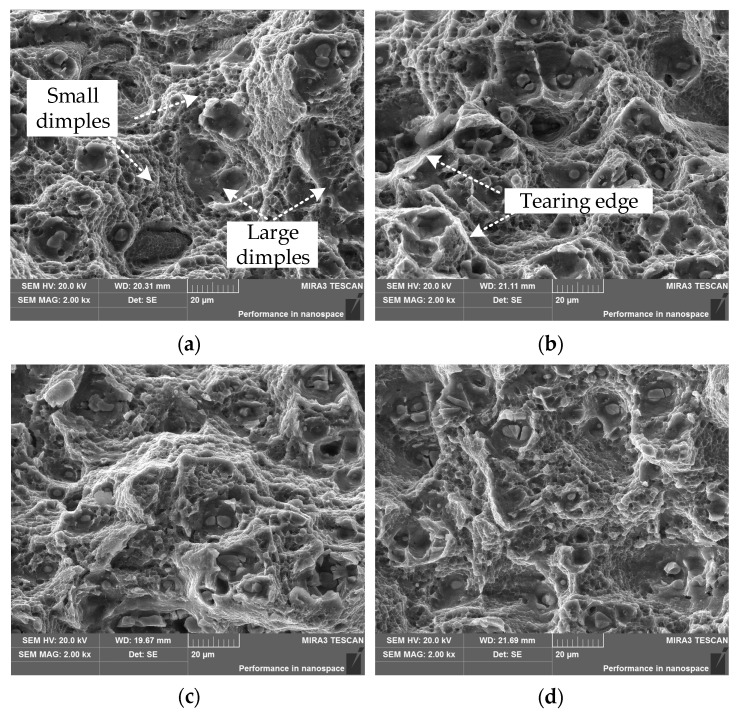
SEM images of fatigue-crack transient region at different creep-aging temperatures: (**a**) 100 °C—9 h; (**b**) 130 °C—9 h; (**c**) 160 °C—9 h; (**d**) 180 °C—9 h.

Figure 6 shows the fracture morphologies of 2524 aluminum alloy in the Fatigue fracture zone (*a* = 25 mm) after aging at different creep-aging temperatures (100 °C, 130 °C, 160 °C, 180 °C) for 9 h. From the fracture surface of the specimen with the creep-aging temperature of 130 °C (see Figure 6b), the large pits caused by the debonding between the precipitation phase and the interface of the aluminum matrix are surrounded by smaller pits. Meanwhile, the large pits are not connected to each other during the fracture process, and there are obvious tearing edges along the small pits, which indicates that the specimen also has good plastic deformation ability. the higher the creep-aging temperature, the larger the dimple size. However, there is no obvious tearing edge, and the fracture morphology is also flatter. The high-temperature creep accelerates the nucleation and growth of the precipitation phase at 180 °C. During the tearing fracture process, the precipitation phase will hinder the dislocation slip due to the incompatibility of the precipitation phase with the aluminum matrix [24], resulting in stress concentration. When the stress concentration exceeds the critical value that the material can withstand, the interface between the precipitate and the aluminum matrix is debonded [25], or the precipitate is fractured, which reduces the plastic deformation ability of the aluminum alloy. With the continuous increase in plastic strain, the interface debonding between the precipitation phase and the aluminum matrix causes the aggregation of large pits. When the effective bearing area is reduced to a critical value, the aluminum matrix will break rapidly.

Under the impact of the creep-aging temperature and the critical size, the enrichment speed of the precipitates are different, and the type, density and length of the precipitates also change [19,26]. In addition, 2524 aluminum alloys under different creep-aging temperature have differences in microstructures and mechanical properties such as yield strength, hardness, and elongation [26,27,28]. Therefore, by controlling the creep-aging temperature, a 2524 aluminum alloy with good plasticity and fatigue properties can be obtained.

### 3.2. Effect of Creep-Aging Stress on Fatigue-Crack Propagation

#### 3.2.1. Effect of Creep Stress on Fatigue-Crack Propagation Rate

**Figure 7:**
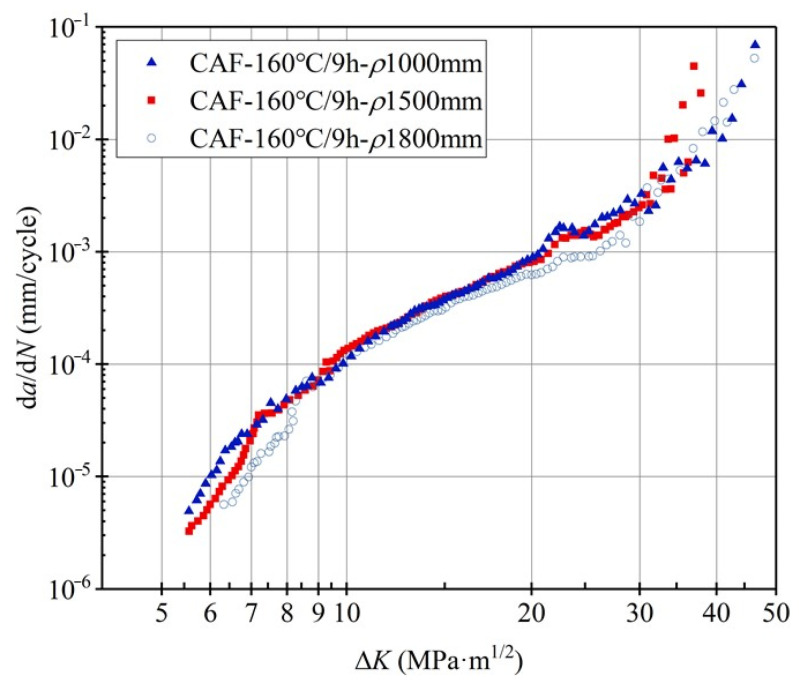
Fatigue-crack propagation rate curves of 2524 aluminum alloy under different creep stresses.

The fatigue-crack propagation rate curves of 2524 aluminum alloy after forming under different creep stresses are shown in Figure 7. In the near-threshold region of fatigue-crack propagation (Δ*K* ≤ 8 MPa·m^1/2^), the fatigue-crack propagation threshold of 2524 aluminum alloy with a forming radius of 1800 mm is higher than that at 1500 mm and 1000 mm, and its propagation rate is also lower than alloys in the other two states. However, the propagation rate with the forming radius of 1000 mm and 1500 mm are relatively close. This shows that the increase in creep stress also reduces the crack propagation resistance of the alloy, but the effect is not as significant as creep-aging temperature. When 8 MPa·m^1/2^ ≤ Δ*K* ≤ 20 MPa·m^1/2^, the crack propagation enters the stable expansion stage, and the curve shows an obvious linear relationship. When Δ*K* exceeds about 18 MPa·m^1/2^, the propagation rate goes up sharply and d*a*/d*N* rapidly expands until fracture occurs.

The linear part in Figure 7 is fitted according to the Paris equation, and the corresponding fitting constants *C*, *n* and the fatigue-crack propagation rate under the same Δ*K* are shown in Table 3. The error value is 4.65%. From the fitting results, the values of the exponents *n* are very close, ranging from 2.7 to 3.0. In the medium stress region and high stress region, the propagation rate of the formed specimens at a radius of 1000 mm is the fastest, indicating that the increase in creep stress reduces the fatigue-crack propagation resistance.

On the one hand, this is because the alloy formed under the radius of 1000 mm has the largest bending deformation and the highest dislocation density contained in the crystal [29]. The large-scale dislocation accumulation and entanglement lead to the work-hardening of the alloy itself. On the other hand, these dislocations provide a large number of nucleation sites, and meanwhile, facilitate the short-circuit expansion of solute atoms in the alloy matrix, promote the precipitation and coarsening of the precipitation phase [30], and indirectly improve the yield strength. In addition, the effect of alloy yield strength on the fatigue-crack propagation rate is mainly reflected in the size of the plastic zone at the crack tip. The plastic zone at the crack tip has the following relationship with the yield strength [31]:

(4) $$r_{p}\left(\alpha \right)=\frac{\Delta K^{2}}{4\pi {\left(\sigma_{0.2}\right)}^{2}}\left(\frac{3}{2}\sin^{2}\alpha +{\left(1-2\mu \right)}^{2}\left(1+\cos\alpha \right)\right)$$

where *r*_p_ is the radius vector; Δ*K* is the stress intensity factor; *σ*_0.2_ is the yield stress; *α* is the polar angle; and *µ* is the Poisson’s ratio of 2524 aluminum alloy. Under the same stress intensity factor amplitude Δ*K*, the size *r*_p_ of the plastic zone at the crack tip is inversely proportional to the yield strength *σ*_0.2_. The larger the *r*_p_, the more energy is absorbed under each cyclic load, and the better the fatigue damage resistance of the alloy (i.e., the increase in the yield strength reduces the crack propagation resistance of the alloy). Therefore, the increase in creep stress has an adverse effect on the fatigue performance of 2524 aluminum alloy.

In different creep-aging specimens, the larger the aging stress, the lower the apparent activation energy, and the lower the resistance encountered by the movement of dislocations [12]. Meanwhile, a large number of dislocations brought by pre-deformation provide a large number of mobile dislocations for creep, and also promote the nucleation of the second phase [32]. Therefore, when the creep specimen is creep formed under larger stress, the precipitation and growth rate of the precipitates are also faster, but the density of the precipitates declines. The enhancement of the pinning effect of precipitation relative to dislocations improves the plastic deformation resistance, and the damage caused by the same fatigue cyclic load is smaller.

The increase in creep stress also reduces the crack propagation resistance relative to unstressed aging. However, compared with the effect of creep-aging temperature, the effect of creep stress is not as significant as that of creep temperature.

#### 3.2.2. Fracture Morphologies at Different Creep Stresses

(1) Fatigue-crack propagation zone

**Figure 8:**
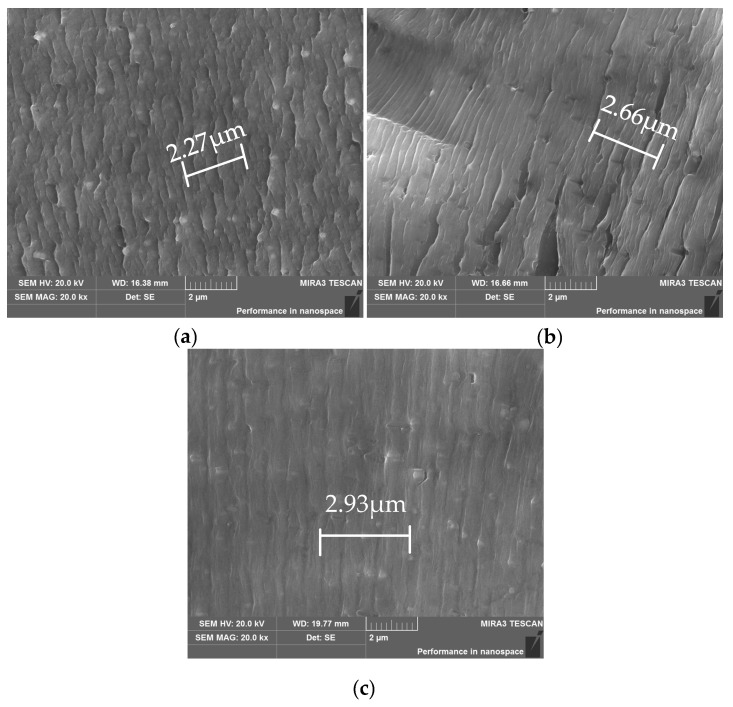
SEM images of fatigue striations under different curvature radii *ρ*: (**a**) *ρ* = 1000 mm; (**b**) *ρ* = 1500 mm; (**c**) *ρ* = 1800 mm.

Figure 8 shows the fatigue striation spacing under the action of different creep curvature radii of 1000 mm, 1500 mm and 1800 mm, respectively. The widths of the five fatigue striations under different creep curvature radii are significantly different, at 2.27 μm, 2.66 μm and 2.93 μm, respectively. Therefore, in the stable propagation stage with the stress intensity factor Δ*K* ranging from 10 to 25 MPa MPa·m^1/2^, the fatigue striation spacing of the specimen with a smaller creep radius of curvature (i.e., a larger creep stress) is larger than that of the specimen with a larger radius of curvature; moreover, the crack propagation rate is faster, and the fatigue resistance is lower.

For 2524 aluminum alloy under stress aging, the preferential growth orientation of the precipitates is sensitive to the applied stress. The applied stress field changes the degree of mismatch between the matrix and the precipitates, which causes the elastic distortion field and the elastic energy of the coherent precipitates to change, thus affecting the precipitation and evolution of the precipitates [33]. Larger aging stress results in lower apparent activation energy, less resistance encountered by the movement of dislocations, and a large number of dislocations caused by pre-deformation; these provide a large number of movable dislocations for creep, and promote the nucleation of the second phase [34]. Therefore, when the creep specimen creeps under the larger stress, the damage caused by the same fatigue cyclic load to the specimen is smaller; however, after the alloy enters the over-aging stage, the fatigue property declines significantly. This is because the precipitation and propagation rate of the precipitation phase goes up with the increase in stress [19], while the density of the precipitation phase goes down with an increase in stress, which makes the plasticity of the aluminum alloy stronger. While the fatigue life of the specimen decreases, the fatigue striation spacing becomes wider.

(2) Fatigue fracture zone

**Figure 9:**
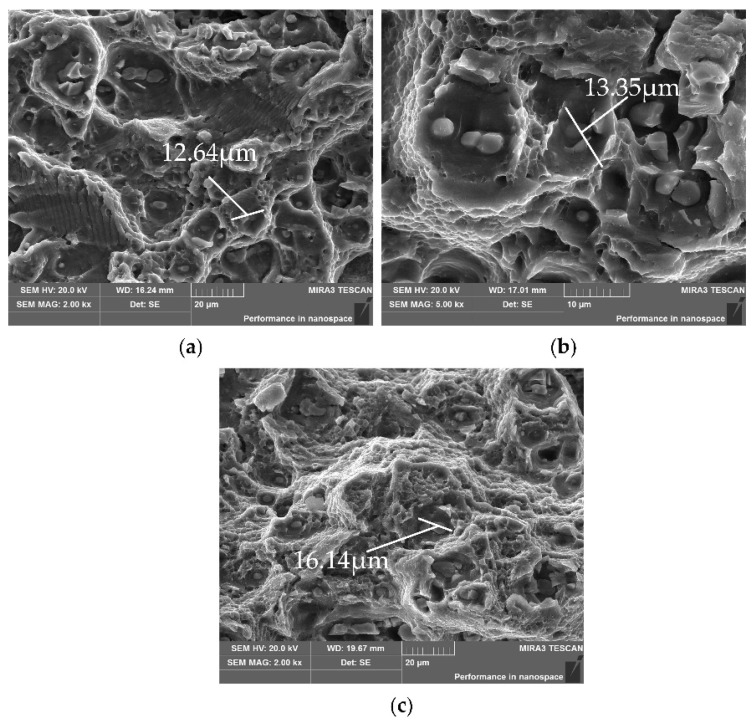
SEM images of the instantaneous break area under different curvature radii *ρ*: (**a**) *ρ* = 1000 mm; (**b**) *ρ* = 1500 mm; (**c**) *ρ* = 1800 mm.

Figure 9 illustrates the SEM images of the fracture zone under different creep stresses, and the curvature radii are 1000 mm, 1500 mm and 1800 mm, respectively. With the increase in creep stress, the fracture morphologies are slightly different; dimples and fractured second-phase particles are clearly seen. In the sample with a curvature radius of 1000 mm, the fracture contains dimples and cleavage fracture morphology. Comparing the fatigue properties of the specimens treated by creep-aging under different curvature radii, under the same fatigue cycle, the specimen with smaller curvature radius has a larger area of the fatigue-crack stable-growth zone, and a smaller area of fracture zone; this demonstrates that the specimen under the action of larger creep stress has longer fatigue stability propagation time and faster instantaneous fracture speed.

The applied creep stress changes the precipitation process of the precipitation phase. Under the action of high stress, the *S* phase is more likely to precipitate and grow. During creep, dislocation density increases and becomes entangled, affecting the dislocation slip. As the creep deformation increases, the dislocations in the grains are rearranged, and the entangled dislocations drive the formation of subgrains [35]. The dislocations entangled with high density form the unit cell-walls of the subgrains, and the dislocation density in the subgrains is low. In addition, the creep stress breaks the balance of the precipitates and changes the precipitation process, and the growth of the precipitates hinders the movement of grain boundaries and dislocations, improving the properties of the aluminum alloy.

## 4. Conclusions

(1) With the same Δ*K*, the crack propagation rate increases with the increase in creep-aging temperature. With the increase in Δ*K*, the difference in crack propagation rate gradually decreases.

(2) Under the same crack length, with the increase in creep-aging temperature, the spacing of fatigue striations increases and the size of dimples decreases, while the number of dimples increases, and the fatigue resistance of the alloy decreases.

(3) In the near-threshold region of fatigue-crack propagation (Δ*K* ≤ 8 MPa·m^1/2^), the fatigue-crack propagation threshold of 2524 aluminum alloy with a forming radius of 1800 mm is higher than that of alloys at 1500 mm and 1000 mm.

**(**4) Under the same fatigue cycle, the specimens under the action of larger creep stress have longer fatigue stable-propagation time and a faster transient fracture speed. However, compared with the effect of creep-aging temperature, the effect of creep stress is not as significant as that of creep temperature. Hence, the creep-aging temperature plays a dominant role in the fatigue-crack propagation of creep-aged 2524 aluminum alloy.

## Figures and Tables

**Table 1:** Chemical composition of 2524 aluminum alloy (mass fraction: wt%).

Cu	Mg	Mn	Fe	Zn	Si	Ti	Cr	Al
4.62	1.32	0.57	0.035	0.004	0.025	0.02	0.001	Bal.

**Table 2:** Paris fitting parameters *C* and *n* at different creep-aging temperatures.

Aging Status	*C*	*n*	d*a*/d*N* = *C*Δ*K^n^*/(mm∙cycle^−1^)		
			Δ*K* = 8	Δ*K* = 12	Δ*K* = 16 (MPa·m^1/2^)
100 °C/9 h	1.77 × 10^−7^	2.99	3.51 × 10^−5^	1.52 × 10^−4^	4.31 × 10^−4^
130 °C/9 h	1.77 × 10^−7^	2.99	4.15 × 10^−5^	1.75 × 10^−4^	3.97 × 10^−4^
160 °C/9 h	4.32 × 10^−7^	2.67	4.52 × 10^−^^5^	2.25 × 10^−4^	4.78 × 10^−4^
180 °C/9 h	2.81 × 10^−7^	2.80	5.02 × 10^−5^	2.13 × 10^−4^	5.74 × 10^−4^

**Table 3:** Paris fitting parameters *C* and *n* of 2524 aluminum alloy under different creep stresses.

*ρ*	*C*	*n*	d*a*/d*N* = *C*Δ*K*^*n*^/(mm·cycle^−1^)			
			Δ*K* = 7	Δ*K* = 12	Δ*K* = 16	Δ*K* = 21 (MPa·m^1/2^)
1000 mm	1.77 × 10^−7^	2.99	2.90 × 10^−5^	2.25 × 10^−4^	4.75 × 10^−4^	1.12 × 10^−^^3^
1500 mm	4.32 × 10^−7^	2.67	2.24 × 10^−^^5^	2.23 × 10^−4^	4.78 × 10^−4^	9.67 × 10^−4^
1800 mm	2.81 × 10^−7^	2.80	1.21 × 10^−5^	2.95 × 10^−4^	4.10 × 10^−4^	6.83 × 10^−4^

## Data Availability

The data presented in this study are available on request from the corresponding authors.
